# Effects of resistance training and aerobic training on improving the composition of middle-aged adults with obesity in an interventional study

**DOI:** 10.1038/s41598-025-11076-w

**Published:** 2025-09-30

**Authors:** Su Hang, Lan Xiaoyu, Wang Jue, Lu Yingli, Zhang Li

**Affiliations:** 1 Department of Physical Education, Kaifeng University, Kaifeng, China; 2 Beijing Institute of Sports Science, Beijing, 100075 China; 3 National Institute of Sports Science, General Administration of Sport, Beijing, 100061 China

**Keywords:** Resistance training, Aerobic training, Obesity, Middle-aged, Fat loss, Obesity, Nutrition, Public health

## Abstract

This study investigated the effectiveness of a resistance and aerobic training model among 71 middle-aged participants aged 30–60 (mean age 44.27 ± 8.67 years; mean BMI 27.94 ± 3.92 kg/m²) with obesity, comprising 36 males and 35 females (male/female ratio ≈ 1.03:1). Participants were categorized into four groups based on their self-reported training regimens: dietary-only (Group C), aerobic fat oxidation (Group F), high-intensity interval training (Group H), and resistance training (Group R). Subjects followed their specialized routines through online and offline sources for at least 12 weeks. Groups F, H, and R demonstrated statistically lower body weight as well as waist-to-hip ratio and body fat percent levels, when assessed against Group C (*P* < 0.01). The combination of resistance training with specific benefits produced larger reductions in waist-to-hip ratio, together with android fat mass, primarily observed among male participants (*P* < 0.01). The participants in Group H demonstrated the greatest decrease in body fat percentage among female subjects (*P* < 0.01), even though Group R participants achieved beneficial results, although their adherence level was less than ideal. Participants from all experimental groups maintained similar levels of muscle mass. The hybrid online and offline approach effectively enhanced adherence and engagement, demonstrating its scalability and potential for managing obesity.

## Introduction

The increasing prevalence of obesity in middle-aged Chinese individuals reflects broader global trends driven by urbanization, reduced physical activity, and poor dietary choices. Obesity is a well-documented risk factor for numerous chronic conditions, including cardiovascular diseases, diabetes, and musculoskeletal disorders^1^. According to the World Obesity Federation, the burden of obesity is rising at an alarming rate globally, with a marked shift toward developing nations, including China^2^. The National Health and Family Planning Commission of China reported that over 50% of Chinese adults have excess body weight, with urbanization and economic growth being key contributors. Studies have shown that individuals with obesity are at greater risk of developing severe complications after contracting COVID-19^3–5^, highlighting the importance of incorporating fat-loss-focused exercise into fitness regimens.

Post-COVID-19 recovery poses significant challenges, including risks of myocarditis from high-intensity aerobic exercise, muscle atrophy, and persistent fatigue, necessitating cautious and structured rehabilitation approaches. High-intensity exercises can exacerbate cardiac abnormalities like myocarditis, emphasizing the need for phased and individualized return-to-exercise protocols^6^. Recent international surveys highlight aerobic exercise as the most widely adopted form of physical activity among diverse populations, reinforcing its global popularity and accessibility^7^. Moreover, contemporary studies affirm the safety and effectiveness of aerobic interventions, even in individuals with chronic conditions, demonstrating improvements in cardiovascular outcomes, body composition, and overall fitness^8–10^. Muscle weakness and atrophy, common in recovering patients, further highlight the importance of light-to-moderate aerobic and resistance training to rebuild strength and reduce fatigue^11,12^. Remote exercise capacity assessments and home-based programs provide safe alternatives for addressing functional impairments while minimizing infection risks^13^. Initiatives like the SPRINTT program demonstrate the efficacy of tailored rehabilitation focusing on aerobic, strength, flexibility, and balance training, particularly for older individuals. Comprehensive exercise-based recovery programs not only restore physical function but also improve respiratory health, fatigue, and cognition, underscoring their role in enhancing quality of life post-COVID-19^14^.

In developing fat loss programs, aerobic training is often prioritized as the primary training method. This typically includes strategies such as maximum fat oxidation intensity exercises and high-intensity aerobic interval training^15^. Supported by substantial evidence of its role in reducing visceral adiposity and improving metabolic health, aerobic exercise remains a cornerstone strategy for obesity management^16^. These findings further justify the inclusion of aerobic modalities as comparators in the present study, alongside resistance and hybrid approaches. However, resistance training, which is a scientifically validated method to enhance muscle strength, increase muscle volume, and improve muscular endurance^17^, has traditionally been relegated to a supplementary role rather than being incorporated as a central component of fat loss program for middle-aged and elderly populations. Resistance training increases muscle mass and elevates resting metabolic rate (RMR), which contributes to higher energy expenditure even at rest^18^. Strength-focused exercises improve balance, agility, and coordination, particularly beneficial for older populations. Resistance exercises stimulate Excess Post-Exercise Oxygen Consumption (EPOC), maintaining an elevated metabolic rate for hours after the activity. The 2018 Global Recommendations on Physical Activity for Good Health by the World Health Organization (WHO) advocates that adults aged 18–64 years, as well as older adults aged 65 and above, should engage in strength-focused activities involving major muscle groups at least twice per week^19^.

Studies underline the efficacy of resistance training in preventing muscle atrophy and enhancing various physical attributes. Resistance training has been shown to significantly improve muscle function through adaptations such as increased muscle fiber cross-sectional area and enhanced motor unit activation, which are crucial for maintaining strength and delaying atrophy^20^. It also effectively promotes recovery from atrophy by activating cellular signaling pathways and increasing the expression of protective proteins^21^.

In addition to preventing muscle loss, resistance training improves physical attributes such as strength, balance, and coordination. Studies have demonstrated significant gains in muscular endurance and flexibility with resistance training regimens, emphasizing its role in comprehensive fitness programs. Furthermore, its ability to enhance basal metabolic rate is linked to improvements in overall energy expenditure and metabolic control, making it a critical program for weight management and metabolic health^22^. Resistance training has also been linked to improved insulin sensitivity, critical for obesity and diabetes management^23^. Furthermore, resistance training is particularly effective in mitigating age-related sarcopenia, making it highly relevant for middle-aged and elderly populations.

As Chinese society gradually reopens, indoor and outdoor fitness venues have resumed operations. However, the SARS-CoV-2 virus remains present, with new variants continually emerging. This poses a significant threat to middle-aged and elderly individuals, making home fitness a safer alternative. This study primarily addresses the following research questions:

1. When fat-loss training for middle-aged individuals with obesity focuses primarily on resistance exercises, can it achieve the goals of weight loss, fat reduction, and muscle gain?
2. What are the comparative advantages of resistance exercise programs versus maximum fat oxidation intensity and high-intensity aerobic interval training programs?
3. Considering the risks of COVID-19 transmission in group settings, can the combination of “offline + online” training modes achieve effective fitness outcomes?

With the easing of restrictions globally and the continuous mutation of the SARS-CoV-2 virus, immunity to the virus declines over time, increasing the likelihood of reinfection. The initial wave of infections in China significantly reduced the physical fitness of many individuals, particularly their cardiopulmonary function. High-intensity aerobic interval training may not be suitable for middle-aged individuals recovering from COVID-19 due to its demanding cardiopulmonary requirements. Instead, moderate-intensity fat oxidation training is a safer and more accessible alternative. However, outdoor gatherings carry infection risks and expose individuals to adverse weather conditions, such as cold and haze. The resistance training program developed in this study, designed with moderate intensity, can be performed at home, minimizing infection risks while restoring muscle strength, improving muscle metabolism and endurance, and reducing fat.

## Research methods

### Research population

The study included 100 middle-aged participants with obesity (46 males and 54 females) who met China’s obesity criteria and were confirmed to have no exercise-related health risks. Participants were aged between 30 and 60 years and provided informed consent before the study. The participants were categorized based on their self-reported primary mode of physical activity over the previous 12 weeks into four groups: the control group (Group C), the maximum fat oxidation intensity group (Group F), the high-intensity aerobic interval training group (Group H), and the resistance exercise group (Group R). A formal power analysis was not performed prior to study commencement, as participants were recruited using a convenience sampling strategy based on availability within the recruitment period. However, the final sample size (*n* = 71) was sufficient to detect significant differences in primary outcomes such as body weight, waist-to-hip ratio, and body fat percentage across intervention groups, as evidenced by the statistical results. All research processes and technical means have passed the ethical review of the Human Exercise Experiment Ethics Committee (approval number: CISSIRB-20191008). All procedures were performed in accordance with the ethical standards of the institutional research committee and with the 1964 Helsinki Declaration and its later amendments or comparable ethical standards.

At baseline, the participants’ mean age ranged from 40.19 ± 9.93 to 47.24 ± 7.74 years across groups, with corresponding 95% CIs between 34.90 and 50.67 years. Mean BMI ranged from 27.30 ± 3.92 to 28.33 ± 5.01 kg/m², with 95% CIs between 25.21 and 30.55 kg/m² (Table 1). These values indicate comparable baseline characteristics among groups.

**Table 1:** Basic information of subjects.

Index	Group C (*n* = 16)	Group F (*n* = 16)	Group H (*n* = 17)	Group R (*n* = 22)	*P*-value
**Age (years)**	40.19 ± 9.93 (95% CI: 34.90–45.48)	46.08 ± 8.28 (95% CI: 41.67–50.49)	43.22 ± 7.92 (95% CI: 39.15–47.29)	47.24 ± 7.74 (95% CI: 43.81–50.67)	0.069
**Height (cm)**	1.65 ± 0.08	1.66 ± 0.10	1.67 ± 0.08	1.67 ± 0.09	0.902
**Weight (kg)**	77.66 ± 12.62 (95% CI: 70.94–84.38)	76.26 ± 16.45 (95% CI: 67.49–85.03)	78.58 ± 16.57 (95% CI: 70.06–87.10)	80.24 ± 20.23 (95% CI: 71.27–89.21)	0.977
**BMI (kg/m²)**	28.19 ± 2.66 (95% CI: 26.77–29.61)	27.30 ± 3.92 (95% CI: 25.21–29.39)	27.92 ± 3.60 (95% CI: 26.07–29.77)	28.33 ± 5.01 (95% CI: 26.11–30.55)	0.89
**Body fat (%)**	36.64 ± 6.86 (95% CI: 32.98–40.30)	34.70 ± 4.62 (95% CI: 32.24–37.16)	35.09 ± 4.89 (95% CI: 32.58–37.60)	36.54 ± 4.82 (95% CI: 34.40–38.68)	0.616
**Physical activity (kcal/day)**	517.80 ± 425.32 (95% CI: 291.16–744.44)	488.13 ± 330.89 (95% CI: 311.81–664.45)	400.86 ± 172.05 (95% CI: 312.40–489.32)	311.68 ± 191.13 (95% CI: 226.94–396.42)	0.139

Note: Group C: male=8, female=8; Group F: male=6, female=10; Group H: male=10, female=7; Group R: male=10, female=12.

### Assessing fat oxidation and aerobic capacity

#### Maximum fat oxidation intensity test

This study assessed fat oxidation and aerobic capacity at a single time point using exercise testing protocols. A standardized treadmill protocol was used to estimate participants’ fat oxidation rates and aerobic performance. Participants wore a gas analyzer breathing mask and a heart rate monitor during the exercise test. After standing quietly on the treadmill for 3 min to establish baseline readings, the treadmill was set to an initial speed of 3 km/h, which was increased by 1 km/h at each subsequent stage. The maximum speed reached was 7 km/h, with each stage lasting 2 min. The exercise test was terminated when one of the following two conditions was met: The respiratory quotient reached 1 and stabilized, indicating a shift away from fat oxidation as the primary energy source; The fat oxidation rate dropped to 0 for more than 1 min. The intensity corresponding to the highest fat oxidation rate observed during the test was designated as the maximum fat oxidation intensity. The heart rate at this point was recorded and used as the target heart rate for subsequent exercises.

#### Maximum oxygen uptake test

The exercise test began with the subject wearing a gas analyzer respirator mask and a heart rate monitor. After standing quietly on the treadmill for 1 min to establish baseline readings, the test commenced at an initial speed of 5 km/h. The treadmill speed increased by 1 km/h every minute until a maximum speed of 12 km/h was reached. Once the maximum speed was achieved, the speed remained constant, and the incline of the treadmill increased by 1% per minute, up to a maximum slope of 5%. The test was terminated, and maximum oxygen uptake (VO_2_​ max) was deemed achieved when two of the following three conditions were met: Oxygen uptake plateaued or decreased despite increasing exercise intensity, defined as a change of less than ± 2 mL/kg/min; Heart rate exceeded the predicted maximum heart rate (220 − age); The respiratory quotient (RQ) exceeded 1.10. The VO_2_​ max recorded during the test was designated as the subject’s maximum oxygen uptake, and the corresponding heart rate at this point was recorded as the target heart rate for subsequent exercise protocols. The equipment used for the test included a German Cortex Metamax 3B gas analyzer, an h/p/cosmos treadmill, and a Polar heart rate monitor.

### Methods of physical indicators

#### Height/weight measurement

Height and weight were measured using a standard stadiometer and a calibrated digital weighing scale. For height measurements, subjects were barefoot and stood upright with their heels, sacrum, and scapulae in contact with the stadiometer column. The head was positioned in the Frankfort horizontal plane, with the subject looking straight ahead. For weight measurements, subjects stood at the center of the scale, ensuring their feet were stable before reading the measurement. To enhance accuracy, subjects wore only light, form-fitting clothing during the measurements.

#### Waist/hip circumference measurement

Waist and hip circumferences were measured with the subject in a standing position after achieving a stable breathing pattern. Waist circumference was measured at the horizontal plane of the umbilicus, while hip circumference was measured at the horizontal plane of the most prominent part of the gluteal region (posteriorly). Measurements were recorded in centimeters (cm), and the waist-to-hip ratio (WHR) was calculated using the formula:

$$\:WHR=\:\frac{\text{W}\text{a}\text{i}\text{s}\text{t}\:\text{C}\text{i}\text{r}\text{c}\text{u}\text{m}\text{f}\text{e}\text{r}\text{e}\text{n}\text{c}\text{e}\:\left(\text{c}\text{m}\right)}{\text{H}\text{i}\text{p}\:\text{C}\text{i}\text{r}\text{c}\text{u}\text{m}\text{f}\text{e}\text{r}\text{e}\text{n}\text{c}\text{e}\:\left(\text{c}\text{m}\right)}$$

#### Body composition determination

Body composition was assessed at the time of study participation. Measurements included the percentage of body fat, total body fat mass, fat mass in the Android region (defined as the area between the superior tangent line of the pelvis and the inferior tangent line of the ribs), and total muscle mass. The dual-energy X-ray absorptiometry (DXA) method, recognized as the gold standard for body composition analysis, was employed for these measurements^24^. The instrument used was the GE Medical System Lunar Prodigy DXA scanner.

### Assessment of dietary intake

At the time of study participation, the resting metabolic rate (RMR) of each subject was measured under fasting conditions during a resting test. Daily physical activity energy expenditure (PAEE) was estimated based on the results of the Chinese Physical Activity Questionnaire (CPAQ) completed by the subjects. The sum of the RMR and PAEE provided the recommended daily energy intake (RDEI) for each subject. For men, the RDEI ranged from 2000 to 2700 kcal, while for women, it ranged from 1600 to 2400 kcal. Personalized dietary guidance was developed based on the “Balanced Dietary Patterns and Food Amounts for Different Energy Requirement Levels” outlined in the 2016 edition of the Dietary Guidelines for Chinese Residents^5^. To ensure adherence, guidance and supervision were conducted online using the Daily Health APP, which allowed for continuous monitoring and individualized feedback throughout the training.

### Training program

Participants were categorized into four groups based on their self-reported primary physical activity behaviors over 12 weeks. Among the four groups, Group C comprised individuals who reported following a dietary plan without participating in any structured exercise. The remaining three groups —F, H, and R—consisted of participants who regularly engaged in aerobic fat oxidation-focused training, high-intensity interval training, and resistance training, respectively. These programs were developed with reference to the American College of Sports Medicine (ACSM) Exercise Testing and Prescription Guidelines^25^. Participants reported exercise behaviors by completing structured surveys that assessed their training frequency as well as session duration and intensity. Participants identified their session format as online, offline, or hybrid so researchers could analyze training delivery methods between demographic groups.

#### Group F

The subjects in Group F performed exercises designed to target their maximum fat oxidation intensity. Each participant wore an exercise bracelet to monitor heart rate during the sessions, allowing real-time tracking and control of exercise intensity. After each session, participants uploaded the complete heart rate record to a designated platform, where researchers evaluated whether the exercise met the prescribed standards. The training frequency was five times per week, with a progressive increase over 12 weeks: Weeks 1–4: 40 min per session; Weeks 5–8: 50 min per session; Weeks 9–12: 60 min per session. The exercise regimen primarily consisted of continuous walking or running and was supplemented with aerobics and swimming to provide variety and ensure adherence.

#### Group H

The subjects in Group H performed structured interval training designed to enhance cardiovascular fitness and fat oxidation. Each participant wore an exercise bracelet to monitor heart rate in real time, ensuring control over exercise intensity. After each session, participants uploaded the complete heart rate record to a designated platform for researchers to evaluate whether the prescribed intensity and duration standards were met. The exercise frequency was five sessions per week, with the primary activities consisting of pedaling and running. The protocol was adjusted progressively over the 12-week period to accommodate the participants’ fitness levels, following the principle of gradual adaptation: Weeks 1–4: Exercise at 80% of VO_2_​max for 4 min, followed by recovery at 50% of VO_2_​max for 3 min. This cycle was repeated for 4 sets per session; Weeks 5–12: Exercise at 90% of VO_2_​max for 4 min, followed by recovery at 60% of VO_2_​max for 3 min. This cycle was repeated for 4 sets per session. This progressive approach ensured that participants were gradually exposed to higher exercise intensities, reducing the risk of injury while maximizing physiological adaptations.

#### Group R

**Fig. 1:**
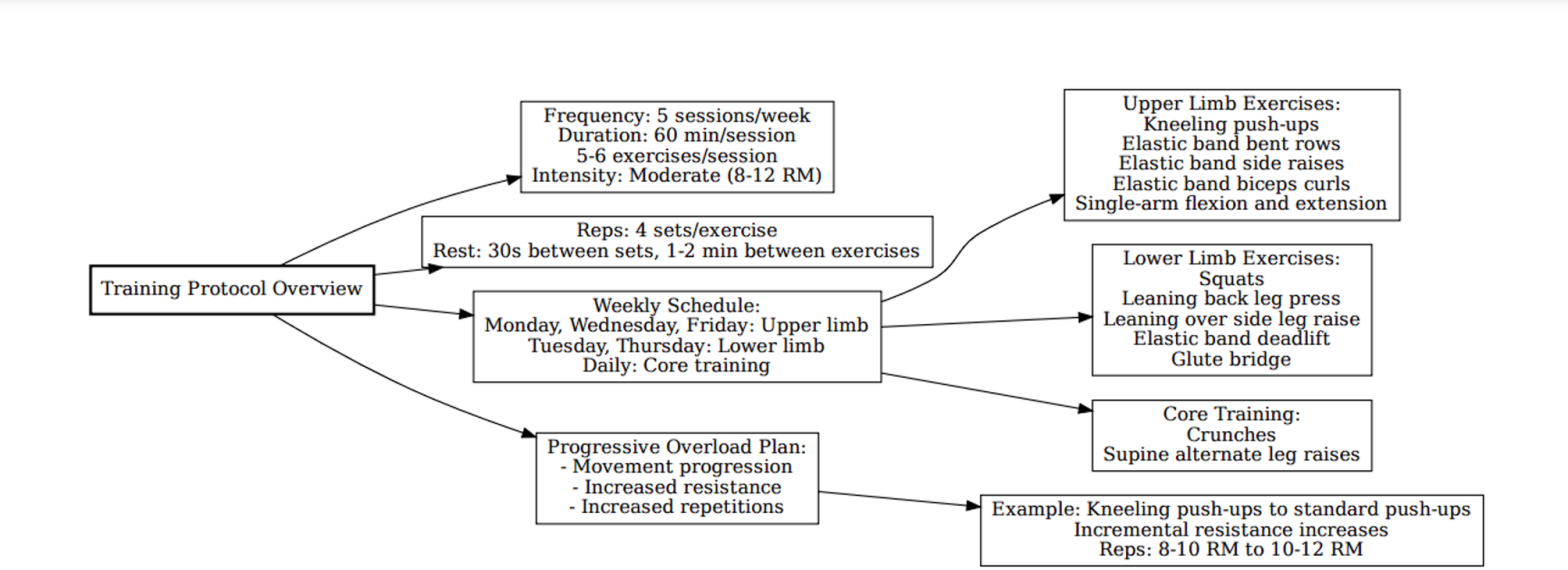
Training protocol.

The resistance training program for Group R was designed based on the principle of alternating upper and lower limb training. The schedule primarily incorporated multi-joint compound movements, such as push-ups, squats, and elastic band deadlifts, to engage multiple muscle groups and maximize energy expenditure per unit time. Additionally, isolated movements using elastic bands, such as biceps curls and single-arm flexion and extension, were included as auxiliary exercises. Each session transitioned from high to low loads to ensure appropriate muscle fatigue management. The progression plan was adjusted weekly to ensure continuous improvement in strength and endurance. Core exercises were excluded from resistance progression and remained consistent throughout the program. An overview of the training protocol is illustrated in Fig. 1, and the advanced plan is given in Table 2.

**Table 2:** Advanced plan for the resistance exercise group.

Time	Number of training sets * number of times	Advanced
1 to 2 weeks	4*(8 ~ 10)	No resistance; auxiliary
3 to 4 weeks	4*(8 ~ 10)	+ 7 lbs elastic band
5 to 6 weeks	4*(11 ~ 12)	+ 7 lbs elastic band
7 to 8 weeks	4*(8 ~ 10)	+ 10 lb elastic band
9 to 10 weeks	4*(11 ~ 12)	+ 10 lb elastic band
11 to 12 weeks	4*12pcs	+ 15 lb elastic band

The participants modified their resistance training activities according to their individual fitness abilities and workout preferences. The female participants reported starting with wall push-ups at low intensity before transitioning to kneeling push-ups based on their comfort and strength development. Participants with higher baseline strength, including males, selected heavier resistance starting with 10-pound elastic bands and then adjusted their workout intensity independently.

### Evaluating the hybrid online and offline training model

This study evaluated the effects of a hybrid online and offline training model across four groups. Participants engaging in physical activity (Groups F, H, and R) reported the format of their training sessions over 12 weeks, indicating whether sessions were conducted online, offline, or through a hybrid approach. Online sessions were conducted using platforms like Tencent Meeting, enabling direct communication with coaches. Self-reported adherence (as a percentage of planned sessions completed) and engagement (rated on a 5-point Likert scale) were used to compare training habits across exercise modalities.

### Data statistics

Statistical analysis was conducted using SPSS 26.0 software. The Shapiro-Wilk test was employed to assess the normality of the data distribution. p-value < 0.05 was considered statistically significant.

## Results

### Changes in body weight and body shape

#### Change in body weight

The results, summarized in Table 3; Fig. 3, showed that the weights of subjects in Groups F, H, and R decreased significantly compared to their baseline weights (*P* < 0.01), while there was no significant change in the weights of subjects in Group C. Significant weight reductions were also observed in males of Groups F, H, and R (*P* < 0.01) compared to baseline. In females, significant weight reductions occurred in Groups F, H, and R, with *P* < 0.01 for Groups F and R and *P* < 0.05 for Group H. When comparing the rate of weight change across groups, the weight reductions in Groups F, H, and R were significantly greater than those in Group C (*P* < 0.05, *P* < 0.01, and *P* < 0.01, respectively), with large to very large effect sizes: Cohen’s d = −0.88 (95% CI: −1.60 to −0.15) for Group F, −1.42 (95% CI: −2.18 to −0.66) for Group H, and − 1.03 (95% CI: −1.72 to −0.35) for Group R. Among males, the weight reductions in the three exercise groups were substantially greater than those in Group C (*P* < 0.01), with very large effect sizes ranging from d = −1.46 to −2.44. In contrast, no significant differences in the rate of weight change were observed among females across groups, and the effect sizes were smaller and their confidence intervals included zero (d = −0.33 to −0.58), indicating more variable responses.

**Table 3:** Changes in body weight of each group before and after training.

	Group C		Group F		Group H		Group *R*	
	Pre-training	Post-training	Pre-training	Post-training	Pre-training	Post-training	Pre-training	Post-training
**Average weight (kg)**	77.66 ± 12.62	77.05 ± 12.97	76.26 ± 16.45	73.08 ± 14.78^b^	78.58 ± 16.57	74.20 ± 15.19^b^	80.24 ± 20.23	76.40 ± 17.77^b^
**Rate of change (%) in overall group**	−0.87 ± 2.55		−3.78 ± 3.95^c^ (d = −0.88, 95% CI: −1.60 to −0.15)		−5.37 ± 3.66^d^ (d = −1.42, 95% CI: −2.18 to −0.66)		−4.36 ± 3.86^d^ (d = −1.03, 95% CI: −1.72 to −0.35)	
**Average weight (kg) – Male**	86.33 ± 6.56	86.36 ± 6.12	91.85 ± 10.37	87.48 ± 10.84^b^	88.49 ± 14.13	82.61 ± 13.97^b^	96.73 ± 17.34	91.18 ± 14.69^b^
**Rate of change (%) – Male**	0.11 ± 2.45		−4.84 ± 1.68^c^ (d = −2.29, 95% CI: −3.64 to −0.93)		−6.72 ± 3.04^d^ (d = −2.44, 95% CI: −3.67 to −1.22)		−5.43 ± 4.58^d^ (d = −1.46, 95% CI: −2.50 to −0.41)	
**Average weight (kg) – Female**	69.00 ± 11.25	67.74 ± 11.16	66.90 ± 11.47	64.43 ± 8.80^b^	64.41 ± 5.90	62.19 ± 6.01^a^	66.50 ± 9.05	64.08 ± 7.87^b^
**Rate of change (%) – Female**	−1.84 ± 2.40		−3.14 ± 4.82 (d = −0.33, 95% CI: −1.27 to 0.61)		−3.44 ± 3.81 (d = −0.51, 95% CI: −1.54 to 0.52)		−3.47 ± 3.07 (d = −0.58, 95% CI: −1.49 to 0.34)	

Note: Change rate = 100% × (after training - before training)/before training; a means *P* < 0.05 compared with before training, b means *P* < 0.01, c means *P* < 0.05 compared with group C, d indicates *P* < 0.01.

### Change in waist-to-hip ratio

**Fig. 2:**
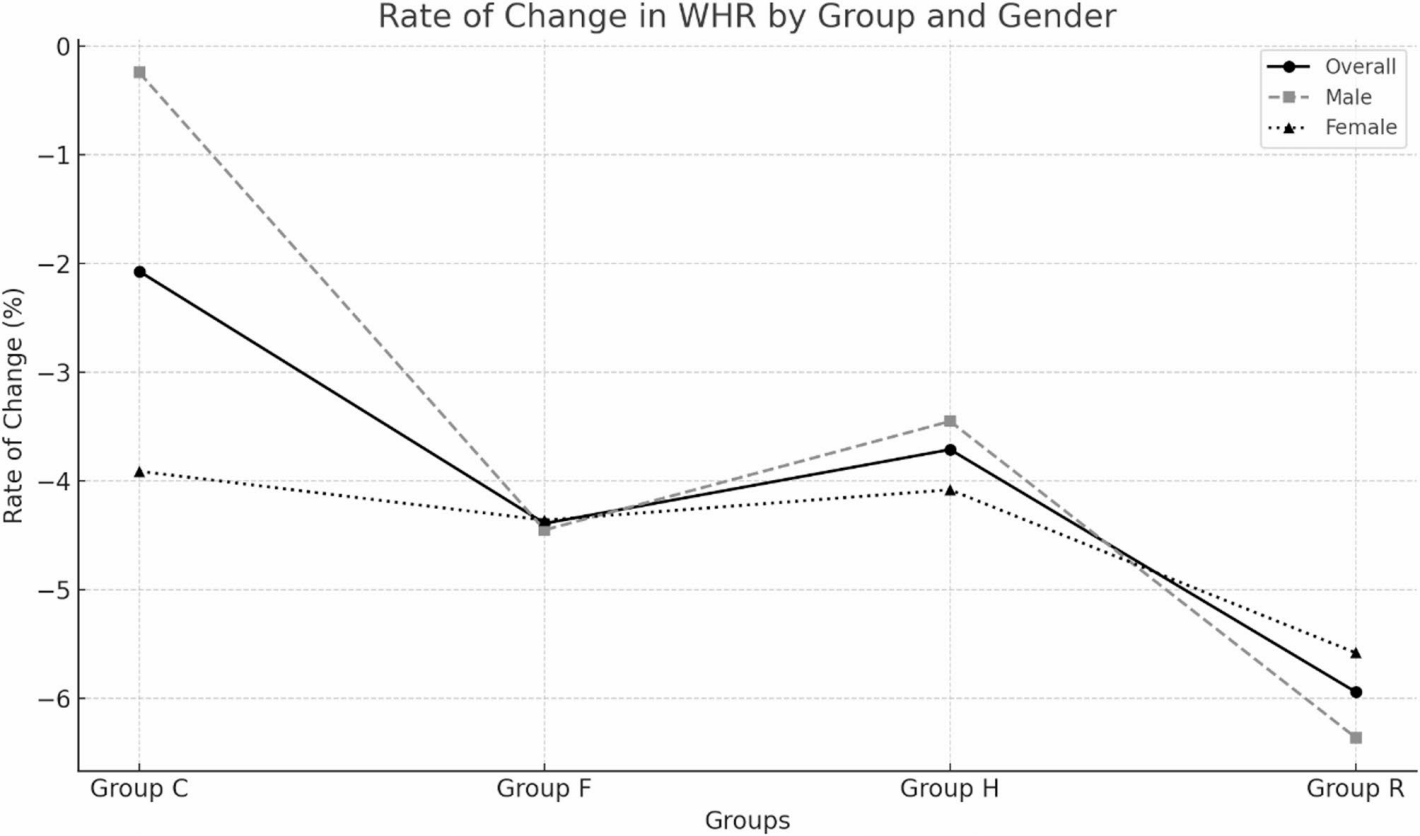
Rate of change in waist-to-hip ratio.

**Fig. 3:**
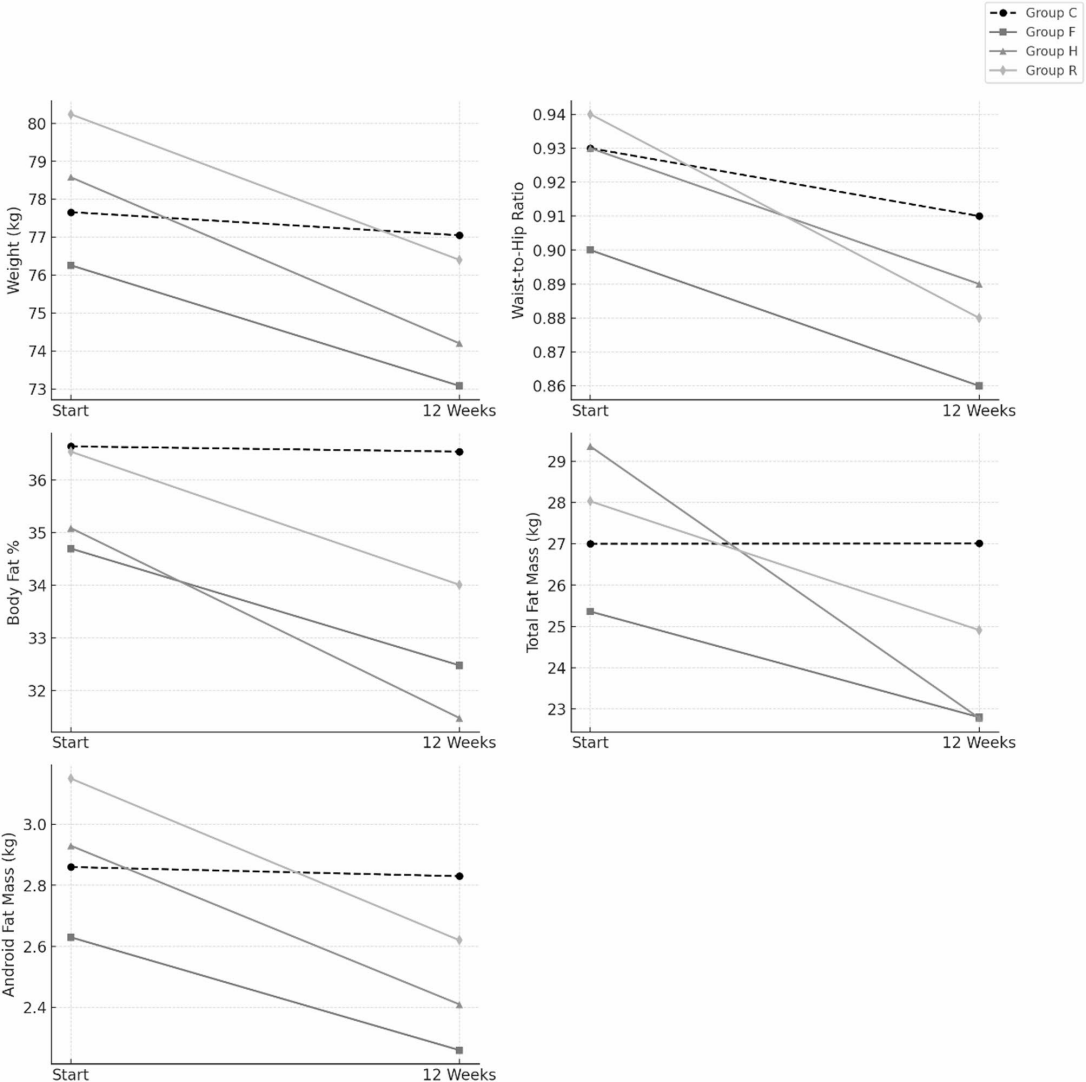
Plot showing changes in weight, waist-to-hip ratio, body fat%, total fat mass, and android fat mass over the 12 weeks across all groups.

The results, as shown in Table 4; Figs. 2 and 3, indicate significant reductions in WHR post-training for subjects in Groups F, H, and R compared to their baseline values (*P* < 0.01), whereas the control group (Group C) showed no significant change. Among the exercise groups, Group R demonstrated the most pronounced reduction in WHR, followed by Group H and Group F. Effect size analyses revealed moderate to large impacts on WHR change compared to control, with Cohen’s d values of −0.49 (95% CI: −1.05 to 0.07) for Group F, −0.63 (95% CI: −1.19 to −0.07) for Group H, and − 0.85 (95% CI: −1.33 to −0.37) for Group R. For male participants, WHR reductions were significant in Groups F, H, and R (*P* < 0.01), accompanied by large effect sizes (d ranging from − 0.89 to −1.17), indicating substantial benefits of these interventions, especially resistance training (Group R), which showed the greatest rate of change. In contrast, for female participants, although significant reductions were also observed in Groups F, H, and R (*P* < 0.05, *P* < 0.01), the effect sizes were smaller and their confidence intervals included zero (d ranging from − 0.10 to −0.75), suggesting more variable intervention effects across exercise modalities in this subgroup. These findings collectively underscore that resistance training (Group R) is particularly effective in reducing WHR, especially in males, with a clear trend of superior outcomes compared to other exercise strategies. The minimal changes observed in the control group further emphasize the necessity of physical activity to achieve meaningful reductions in WHR.

**Table 4:** Changes in the waist-to-hip ratio of subjects before and after training.

	Group C		Group F		Group H		Group *R*	
	Pre-training	Post-training	Pre-training	Post-training	Pre-training	Post-training	Pre-training	Post-training
**Average waist-to-hip ratio**	0.93 ± 0.06	0.91 ± 0.06	0.90 ± 0.06	0.86 ± 0.06^b^	0.93 ± 0.05	0.89 ± 0.06^b^	0.94 ± 0.09	0.88 ± 0.07^b^
**Rate of change (%) in overall group**	−2.07 ± 2.92		−4.39 ± 5.94 (d = −0.49, 95% CI: −1.05 to 0.07)		−3.71 ± 2.70 (d = −0.63, 95% CI: −1.19 to −0.07)		−5.94 ± 5.05^c^ (d = −0.85, 95% CI: −1.33 to −0.37)	
**Average waist-to-hip ratio in males**	0.95 ± 0.05	0.95 ± 0.04	0.95 ± 0.02	0.91 ± 0.06^a^	0.97 ± 0.03	0.93 ± 0.05^a^	1.00 ± 0.09	0.93 ± 0.06^b^
**Rate of change (%) in males**	−0.24 ± 2.40		−4.45 ± 5.84 (d = −0.89, 95% CI: −2.03 to 0.25)		−3.45 ± 3.38 (d = −1.02, 95% CI: −1.94 to −0.10)		−6.36 ± 6.10^d^ (d = −1.17, 95% CI: −2.14 to −0.20)	
**Average waist-to-hip ratio in females**	0.91 ± 0.06	0.87 ± 0.05^a^	0.87 ± 0.05	0.83 ± 0.04^b^	0.88 ± 0.04	0.84 ± 0.04^a^	0.89 ± 0.07	0.84 ± 0.06^b^
**Rate of change (%) in females**	−3.91 ± 2.21		−4.36 ± 6.31 (d = −0.10, 95% CI: −1.02 to 0.82)		−4.08 ± 1.45 (d = −0.75, 95% CI: −1.83 to 0.33)		−5.58 ± 4.22 (d = −0.48, 95% CI: −1.30 to 0.34)	

Note: Change rate = 100% × (after training - before training)/before training; compared with before training, a means *P* < 0.05, b means *P* < 0.01; c means compared with group C, *P* < 0.05, d means *P* < 0.01.

## Body composition changes

### Body fat percentage

**Fig. 4:**
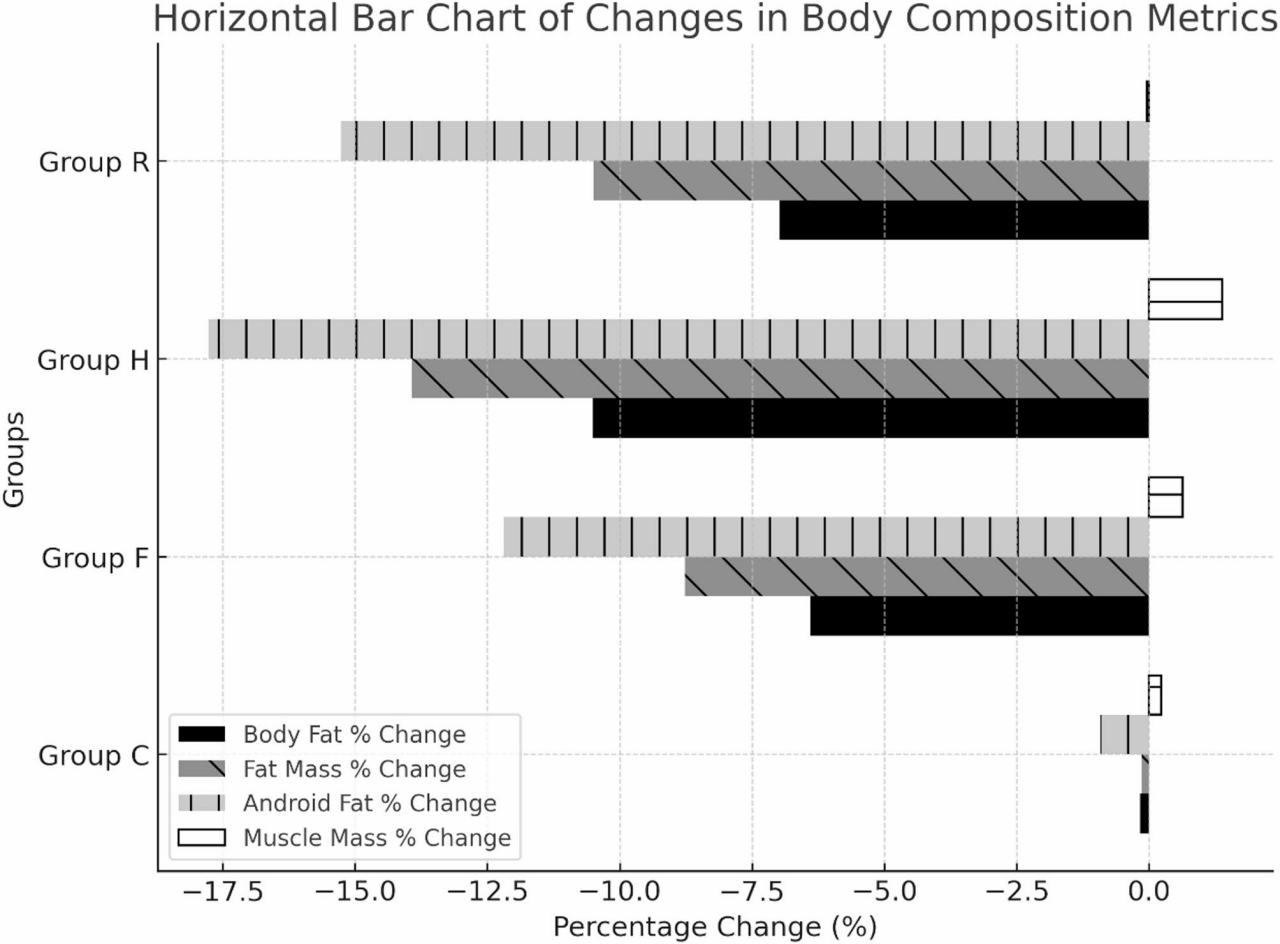
Changes in body composition metrics.

As shown in Table 5; Figs. 3 and 4, the body fat percentage of subjects in groups F, H, and R was significantly lower after the training compared to baseline (*P* < 0.01). Specifically, the body fat percentage of men in groups F, H, and R showed a significant reduction compared to baseline (*P* < 0.01). For women, the body fat percentage in groups F, H, and R also decreased significantly compared to baseline (*P* < 0.05 for group F; *P* < 0.01 for groups H and R). Analysis of the rate of change in body fat percentage between groups revealed that the reduction in groups F, H, and R was significantly greater than in group C (*P* < 0.01). Effect size analyses further demonstrated large to very large intervention effects, with Cohen’s d = −1.11 (95% CI: −1.86 to −0.37) for Group F, −2.43 (95% CI: −3.33 to −1.53) for Group H, and − 1.76 (95% CI: −2.52 to −1.00) for Group R.

Furthermore, the reduction in group H was significantly greater than in groups F and R (*P* < 0.05). Among males, effect sizes indicated very large impacts in all intervention groups (d = −2.32 to −2.43), highlighting robust reductions in body fat percentage compared to controls. Gender-specific analyses showed that the changes in body fat percentage for men in groups F, H, and R were significantly greater than for men in group C (*P* < 0.01). Among women, the changes in body fat percentage in groups H and R were significantly different from those in group C (*P* < 0.01 for group H; *P* < 0.05 for group R). The corresponding effect sizes showed a very large impact in Group H (d = −2.38) and a large effect in Group R (d = −1.52), while Group F exhibited a smaller, non-significant effect (d = −0.49, 95% CI crossing zero), suggesting more modest improvements. Additionally, the reduction in group H was significantly greater than in groups F and R for women (*P* < 0.01 and *P* < 0.05, respectively).

**Table 5:** Changes in body fat percentage of subjects before and after training.

	Group C		Group F		Group H		Group *R*	
	Pre-training	Post-training	Pre-training	Post-training	Pre-training	Post-training	Pre-training	Post-training
**Average body fat percentage (%)**	36.64 ± 6.86	36.54 ± 6.90	34.70 ± 4.62	32.48 ± 4.78^b^	35.09 ± 4.89	31.48 ± 5.15^b^	36.54 ± 4.82	34.01 ± 4.89^b^
**Rate of change (%) in overall group**	−0.17 ± 3.63		−6.39 ± 7.02^d^ (d = −1.11, 95% CI: −1.86 to −0.37)		−10.51 ± 4.77^d, e^ (d = −2.43, 95% CI: −3.33 to −1.530)		−6.98 ± 4.03^d, g^ (d = −1.76, 95% CI: −2.52 to −1.00)	
**Average body fat percentage (%) in males**	31.43 ± 5.03	31.60 ± 4.76	32.12 ± 3.27	28.47 ± 4.21^b^	43.04 ± 4.95	29.57 ± 5.72^b^	34.52 ± 4.92	31.54 ± 4.74^b^
**Rate of change (%) in males**	0.71 ± 3.52		−11.54 ± 7.06^d^ (d = −2.32, 95% CI: −3.68 to −0.95)		−10.89 ± 5.55^d^ (d = −2.43, 95% CI: −3.66 to −1.21)		−8.58 ± 5.03^d^ (d = −2.10, 95% CI: −3.25 to −0.94)	
**Average body fat percentage (%) in females**	41.85 ± 3.66	41.49 ± 4.87	36.25 ± 4.75	34.89 ± 3.31^a^	38.03 ± 3.16	34.20 ± 2.64^b^	48.23 ± 4.22	36.08 ± 4.14^b^
**Rate of change (%) in females**	−1.04 ± 3.77		−3.30 ± 5.12 (d = −0.49, 95% CI: −1.44 to 0.45)		−9.96 ± 3.73^d, f^ (d = −2.38, 95% CI: −3.70 to −1.05)		−5.64 ± 2.45^c, g^ (d = −1.52, 95% CI: −2.53 to −0.51)	

Note: Change rate = 100% × (after training - before training)/before training; compared with before training, a means *P* < 0.05, b means *P* < 0.01; c means *P* < 0.05 compared with group C, d means *P* < 0.01; e means *P* < 0.05 compared with F group, f means *P* < 0.01; g means *P* < 0.05 compared with H group.

### Changes in total body fat mass

As shown in Table 6; Figs. 3 and 4, the total body fat mass of subjects in groups F, H, and R was significantly lower after the training compared to baseline (*P* < 0.01). Specifically, the total body fat mass of men in groups F, H, and R showed a significant reduction compared to baseline (*P* < 0.01). For women, total body fat mass in groups F, H, and R also decreased significantly (*P* < 0.05 for group F; *P* < 0.01 for groups H and R).

Analysis of the rate of change in total body fat mass between groups revealed that the reduction in groups F, H, and R was significantly greater than in group C (*P* < 0.05 for group F; *P* < 0.01 for groups H and R). Effect size analyses confirmed these findings, indicating large to very large intervention impacts with Cohen’s d = −1.06 (95% CI: −1.80 to −0.33) for Group F, −2.22 (95% CI: −3.09 to −1.35) for Group H, and − 1.73 (95% CI: −2.43 to −1.02) for Group R. The rate of total body fat mass across the three exercise groups was also significantly higher than in group C (*P* < 0.01). Among males, effect sizes were exceptionally large across interventions (d = −2.63 to −3.03), highlighting robust reductions, while among females, the strongest effects were seen in Groups H and R (d = −1.53 and − 1.15, respectively). The effect in Group F among females was smaller and its CI included zero (d = −0.39), indicating more variable outcomes. Additionally, among men and women in groups F and R, the reduction in total body fat mass was significantly greater for men compared to women within the same groups (*P* < 0.05).

**Table 6:** Changes in the total body fat mass of subjects before and after training.

	Group C		Group F		Group H		Group *R*	
	Pre-training	Post-training	Pre-training	Post-training	Pre-training	Post-training	Pre-training	Post-training
**Average total body fat mass (kg)**	27 ± 6.43	27.01 ± 6.91	25.36 ± 6.57	22.80 ± 4.99^b^	9.36 ± 7.30	22.78 ± 7.30^b^	28.03 ± 8.00	24.91 ± 6.58^b^
**Rate of change (%) in overall group**	−0.14 ± 5.39		−8.77 ± 9.55^c^ (d = −1.06, 95% CI: −1.80 to −0.33)		−13.93 ± 7.01^d^ (d = −2.22, 95% CI: −3.09 to −1.35)		−10.49 ± 5.98^d^ (d = −1.73, 95% CI: −2.43 to −1.02)	
**Average total body fat mass (kg) in males**	26.18 ± 5.77	26.55 ± 5.49	28.43 ± 5.18	24.25 ± 5.45^b^	28.40 ± 8.68	24.19 ± 9.02^b^	32.50 ± 8.96	28.04 ± 7.88^b^
**Rate of change (%) in females**	1.69 ± 5.03		−15.07 ± 6.74^d, e^ (d = −3.03, 95% CI: −4.83 to −1.23)		−15.76 ± 7.30^d^ (d = −3.01, 95% CI: −4.50 to −1.51)		−13.55 ± 6.62^d, e^ (d = −2.63, 95% CI: −3.90 to −1.35)	
**Average total body fat mass (kg) in females**	27.85 ± 7.34	27.47 ± 8.47	23.52 ± 6.86	21.93 ± 4.78^a^	23.43 ± 3.49	20.77 ± 3.43^b^	24.31 ± 4.81	22.30 ± 3.95^b^
**Rate of change (%) in females**	−1.98 ± 5.41		−4.99 ± 9.19 (d = −0.39, 95% CI: −1.40 to 0.62)		−11.33 ± 6.13 (d = −1.53, 95% CI: −2.65 to −0.41)		−7.94 ± 4.11 (d = −1.15, 95% CI: −2.00 to −0.30)	

Note: Change rate = 100% × (after training - before training)/before training; a means *P* < 0.05 compared with before training, b means *P* < 0.01; c means *P* < 0.05 compared with group C, d means *P* < 0.01; e means *P* < 0.05 compared with women in the same group.

### Android area fat mass changes

As shown in Table 7; Figs. 3 and 4, the results obtained using repeated measures ANOVA indicate that the fat mass in the android region of subjects in groups F, H, and R was significantly lower after the training compared to baseline (*P* < 0.01). Specifically, the fat mass in the android region for men in groups F, H, and R was significantly reduced compared to the baseline (*P* < 0.01). For women, fat mass in the android region in groups F, H, and R also decreased significantly (*P* < 0.05 for groups F and H; *P* < 0.01 for group R).

Analysis of the rate of change in android region fat mass revealed that the reduction in groups F, H, and R was significantly greater than in group C (*P* < 0.01). Effect size analyses further confirmed these findings, showing large to very large intervention impacts with Cohen’s d = −1.23 (95% CI: −2.01 to −0.45) for Group F, −2.15 (95% CI: −3.04 to −1.26) for Group H, and − 1.61 (95% CI: −2.34 to −0.89) for Group R. Among these, the decreases in groups F, H, and R were significantly higher than in group C (*P* < 0.01), with reductions in groups H and R being greater than in group C (*P* < 0.05). In subgroup analyses, effect sizes in males were very large across interventions (d ranging from − 1.81 to −2.61), while in females the strongest effects were observed in Groups H and R (d = −1.36 and − 1.11, respectively); the effect in Group F was more modest and its confidence interval included zero (d = −0.63), indicating less consistent outcomes in this subgroup.

**Table 7:** Changes in fat mass in the android region of subjects before and after training.

	Group C		Group F		Group H		Group *R*	
	Pre-training	Post-training	Pre-training	Post-training	Pre-training	Post-training	Pre-training	Post-training
**Average android fat mass (kg)**	2.86 ± 0.82	2.83 ± 0.87	2.63 ± 0.82	2.26 ± 0.63^b^	2.93 ± 1.16	2.41 ± 1.01^b^	3.15 ± 1.32	2.62 ± 0.99^b^
**Rate of change (%) in overall group**	−0.92 ± 8.64		−12.19 ± 10.46^d^ (d = −1.23, 95% CI: −2.01 to −0.45)		−17.76 ± 7.59^d^ (d = −2.15, 95% CI: −3.04 to −1.26)		−15.26 ± 8.93^d^ (d=−1.61, 95% CI: −2.34 to −0.89)	
**Average android fat mass (kg) in males**	3.18 ± 0.81	3.15 ± 0.75	3.23 ± 0.61	2.65 ± 0.63^b^	3.49 ± 1.21	2.80 ± 1.15^b^	4.13 ± 1.29	3.34 ± 1.02^b^
**Rate of change (%) in males**	−0.33 ± 7.87		−18.30 ± 6.05^d^ (d = −2.53, 95% CI: −4.10 to −0.96)		−20.55 ± 6.90^d^ (d = −2.61, 95% CI: −3.97 to −1.25)		−18.38 ± 10.09^d^ (d = −1.81, 95% CI: −3.02 to −0.60)	
**Average android fat mass (kg) in females**	2.53 ± 0.73	2.52 ± 0.91	2.27 ± 0.74	2.03 ± 0.53^a^	2.13 ± 0.33	1.84 ± 0.33^a^	2.34 ± 0.60	2.01 ± 0.40^b^
**Rate of change (%) in females**	−1.51 ± 9.86		−8.53 ± 11.05 (d = −0.63, 95% CI: −1.68 to 0.42)		−13.77 ± 7.12^c^ (d = −1.36, 95% CI: −2.50 to −0.22)		−12.66 ± 7.27^c^ (d = −1.11, 95% CI: −2.02 to −0.20)	

Note: Δ = 100% × (after training – before training)/before training; a means *P* < 0.05 compared with before training, b means *P* < 0.01; c means *P* < 0.05 compared with group C, d means *P* < 0.01.

### Changes in total body muscle mass

The results indicate varied changes in total body muscle mass across the groups post-training (Table 8; Fig. 4). In Group C, there was a minimal increase in total body muscle mass, with no significant differences observed between pre- and post-training values. In Group F, muscle mass increased slightly, while Group H demonstrated the highest average increase in muscle mass, particularly for females, who showed a significant increase (*P* < 0.01). Conversely, Group R exhibited a slight decrease in muscle mass overall. Effect size analyses supported these findings, revealing negligible to small impacts in overall rates of change, with Cohen’s d ranging from − 0.11 to 0.30 and all confidence intervals crossing zero, indicating a lack of robust group-level effects.

Gender-specific findings revealed that male participants in Groups F and C experienced modest increases in muscle mass, while those in Group H showed a decline in muscle mass (*P* < 0.05), and males in Group R experienced a more pronounced reduction. Consistent with this, effect sizes in males were generally small to moderate, with wide confidence intervals overlapping zero (d = −0.98 to 0.14), suggesting no clear intervention advantage. Among female participants, Group H showed the most notable increase in muscle mass, suggesting that high-intensity aerobic interval training may be particularly beneficial for increasing muscle mass in females. This was reflected in a Cohen’s d of 0.97 for Group H females, although the confidence interval included zero (95% CI: −0.20 to 2.14), emphasizing the need for cautious interpretation. The small changes in Group R highlight potential limitations in resistance training’s efficacy for maintaining or increasing muscle mass under the conditions studied.

**Table 8:** Changes in the subject’s whole body muscle mass.

	Group C		Group F		Group H		Group *R*	
	Pre-training	Post-training	Pre-training	Post-training	Pre-training	Post-training	Pre-training	Post-training
**Average total body muscle mass (kg)**	47.27 ± 10.17	47.42 ± 10.47	47.69 ± 11.09	48.01 ± 11.62	48.78 ± 10.33	49.22 ± 9.60	48.81 ± 13.03	48.62 ± 12.35
**Rate of change (%) in overall group**	0.22 ± 2.46		0.63 ± 3.98 (d = 0.12, 95% CI: −0.52 to 0.76)		1.38 ± 4.67 (d = 0.30, 95% CI: −0.37 to 0.97)		−0.05 ± 2.79 (d = −0.11, 95% CI: −0.64 to 0.42)	
**Average total body muscle mass (kg) in males**	56.51 ± 3.17	56.94 ± 3.22	59.78 ± 6.47	60.63 ± 8.07	56.28 ± 5.54	55.89 ± 6.12	60.43 ± 9.06	59.65 ± 8.50
**Rate of change (%) in males**	0.78 ± 2.11		1.26 ± 4.80 (d = 0.14, 95% CI: −1.01 to 1.29)		−0.78 ± 2.29^b^ (d = −0.71, 95% CI: −1.71 to 0.29)		−1.19 ± 2.38 (d = −0.98, 95% CI: −2.00 to 0.04)	
**Average total body muscle mass (kg) in females**	38.02 ± 4.03	37.89 ± 4.12	40.43 ± 5.06	40.44 ± 4.36	38.07 ± 3.35	39.70 ± 3.11^a^	12/39 ± 5.64	39.44 ± 5.43
**Rate of change (%) in females**	−0.34 ± 2.79		0.24 ± 3.62 (d = 0.19, 95% CI: −0.81 to 1.19)		4.47 ± 5.61 (d = 0.97, 95% CI: −0.20 to 2.14)		0.91 ± 2.84 (d = 0.45, 95% CI: −0.44 to 1.34)	

Note: Rate of change = 100% × (post-training - pre-training)/pre-training; Compared to pre-training, a indicates *P* < 0.01 b means compared with women in the same group, *P* < 0.05.

### Online session adherence and engagement

Table 9 provides a comprehensive analysis of the impact of training on body weight and body fat percentage across all groups, along with insights into adherence and engagement for online sessions in Groups F, H, and R. Effect size analyses reinforced these findings, revealing progressively larger impacts on body fat percentage across intervention groups: Cohen’s d = −0.50 for Group F, −0.56 for Group H, and − 0.80 for Group R, indicating moderate to large effects. Group R, which underwent the hybrid resistance training program, exhibited the highest mean weight reduction (4.47 kg) and body fat percentage reduction (4.01%), both statistically significant (*p* < 0.001). Although weight changes were smaller in magnitude (d = −0.21 to −0.30 across F, H, and R), the corresponding reductions in body fat highlight the qualitative shifts in body composition.

Group F, focusing on aerobic training, and Group H, using interval training, also demonstrated substantial reductions in weight and body fat, with average adherence rates of 90.16% and 87.95%, respectively, and high engagement scores (~ 4.42–4.46). In contrast, Group C, which participated only in the dietary plan, showed minimal and statistically insignificant changes in weight and body fat (Cohen’s d close to zero for both metrics), highlighting the limited impact of diet alone without exercise. Notably, Group R achieved its success despite slightly lower adherence (89.73%), underscoring the effectiveness of the hybrid resistance training model combined with consistent participant engagement. Overall, these results emphasize that moderate to large effect sizes on body fat reduction were achieved even with only modest impacts on weight, underscoring the importance of structured exercise interventions. The data underscores the critical role of structured exercise plans, especially those blending offline and online modalities, in achieving significant physiological improvements.

**Table 9:** Results of online session adherence and engagement for all groups.

Group	Weight Mean (Pre)	Weight Mean (Post)	Weight *p*-value	Weight (kg) change (mean Δ, d, CI)	Body Fat Mean (Pre)	Body Fat Mean (Post)	Body Fat *p*-value	Body fat (%) change (mean Δ, d, CI)	Avg. Online Session Adherence (%)	Avg. Online Session Engagement Score
C	70.67	69.67	0.12	−1.0 (d=−0.07, 95% CI: −0.52 to 0.38)	27.63	28.24	0.29	+ 0.6 (d = + 0.12, 95% CI: −0.34 to 0.58)	NA	NA
F	75.14	72.05	0.00	−3.1 (d=−0.21, 95% CI: −0.67 to 0.25)	30.00	27.48	0.00	−2.5 (d=−0.50, 95% CI: −1.00 to 0.00)	90.16	4.46
H	73.24	70.53	0.00	−2.7 (d=−0.18, 95% CI: −0.63 to 0.27)	28.86	26.09	0.00	−2.8 (d=−0.56, 95% CI: −1.06 to −0.06)	87.95	4.42
R	72.18	67.71	0.00	−4.5 (d=−0.30, 95% CI: −0.71 to 0.11)	29.00	24.99	0.00	−4.0 (d=−0.80, 95% CI: −1.26 to −0.34)	89.73	4.45

## Discussion

### Effect of resistance training versus aerobic training on weight and body shape in middle-aged people with obesity

In this study, there was a significant reduction in body weight in both the resistance training and aerobic training groups, suggesting that resistance training can also play a significant role in weight reduction. Previous studies have demonstrated that resistance training alone is effective in improving fat metabolism in individuals with obesity^26,27^. Specifically, moderate-intensity resistance training at 8–12 RM over approximately 12 weeks has been shown to yield significant results^28^, which is consistent with the findings of this study. Additionally, resistance training has been found to be particularly effective in reducing abdominal fat^29^ and is associated with a lower likelihood of weight regain, facilitating long-term weight control^30^.

This study underscores the significant impact of resistance training on body composition, with particular benefits observed in middle-aged individuals with obesity. Resistance training resulted in notable reductions in waist and hip circumferences and waist-to-hip ratio, surpassing the effects of low-intensity aerobic training, especially among men. Such outcomes align with findings from Seo et al. (2023), which demonstrated significant improvements in body composition and muscle function through resistance training in middle-aged women with obesity^31^. Similarly, Isenmann et al. (2023) found that resistance training effectively alters body composition, although responses can vary based on menopausal status in women^32^.

The greater efficacy of resistance training in reducing abdominal fat and waist-to-hip ratio in men may be attributed to sex-specific physiological differences. As shown in Mahan et al. (2019), men generally exhibit more significant improvements in lean body mass and fat reduction compared to women following resistance training^33^. This is partly due to their higher muscle mass, which enhances fat oxidation during training. Furthermore, the targeted engagement of large muscle groups, such as the lower back and gluteal regions, contributes to stronger core muscles, a feature less prominent in aerobic exercises.

This study provides compelling evidence that resistance training surpasses low-intensity aerobic exercise in reducing waist and hip circumferences among men under equivalent caloric intake and exercise load conditions. This finding is consistent with the broader body of research demonstrating the metabolic and cardiovascular benefits of resistance training. Studies indicate that resistance training improves muscle mass, enhances glucose metabolism, and reduces risk factors for metabolic syndrome, such as insulin resistance and dyslipidemia^34,35^. Additionally, resistance training effectively lowers cholesterol levels and improves lipid profiles, contributing to cardiovascular disease prevention^36^. It also enhances muscular strength and endurance, making it a cornerstone for cardiac rehabilitation and overall cardiovascular health improvement^37^. The ability of resistance training to engage larger muscle groups and boost energy expenditure underscores its effectiveness in reducing abdominal fat and supporting long-term metabolic health^38^. These findings highlight resistance training as a superior program for preventing and managing cardiovascular and metabolic diseases.

These findings support the larger body of literature that has shown the increased importance of exercise interventions in enhancing anthropometric measures, body composition, psychological state, physical functioning, and cardiometabolic fitness of individuals with obesity^39–42^. Recent investigations have consistently demonstrated that aerobic, resistance, and combined training exercise modalities substantially decrease body weight and fat mass and also improve metabolic profiles, reduce blood pressure, improve insulin sensitivity, and improve mood and quality of life. Moreover, high-intensity intervention or the hybrid protocol performed particularly significantly well in maximizing cardiovascular and metabolic outcomes^43–46^. This highlights the multidimensional advantages of exercise planning beyond weight loss and validates the significance of numeric training strategies in correcting excess body mass and related health risks.

### Effect of resistance training on the composition of middle-aged people with obesity

In this study, the body fat content of the resistance training group was significantly reduced following the exercise routine. Relevant studies have shown that both resistance and aerobic training improve body composition in individuals with obesity^47^, consistent with the findings of this study. Resistance training has been shown to reduce body fat and enhance muscle cross-sectional area, particularly when combined with adequate protein intake^48^. Similarly, aerobic training is effective in decreasing body fat and improving cardiorespiratory fitness, while resistance training excels in building muscle strength and explosivity^49^.

The combination of resistance and aerobic training appears to yield superior results for body composition improvements, with studies highlighting their complementary effects. For example, concurrent training significantly reduced body fat percentage and improved BMI in females with obesity^50^. Moreover, integrated training approaches addressing both aerobic and resistance modalities have been linked to enhanced metabolic outcomes and fat mass reduction in obesity^51^. Aerobic and resistance training improves body composition through distinct mechanisms, including enhanced energy expenditure, fat oxidation, and muscle hypertrophy^52^.

Resistance training also enhances muscle mass and volume, elevates basal metabolic rate, and promotes fat burning^53^. Additionally, resistance training has been shown to improve insulin sensitivity and enhance glucose utilization in skeletal muscles, thereby increasing energy expenditure in patients with diabetes^54,55^. This study demonstrated that resistance training effectively reduced fat content in the Android region and improved abdominal obesity. Supporting this, Zelber-Sagi et al. found that resistance training significantly reduced trunk fat and android fat in individuals with non-alcoholic fatty liver disease, aligning with the results of this study^56^.

Moreover, the issue of age-related anabolic resistance, which is the reduced protein synthesis response to dietary amino acids and to resistance exercise among elderly muscle^57–60^, may also contribute to the observed few hypertrophic responses in this population. These together with the moderate intensities of training adopted in this study that proved to be statistically insignificant in triggering muscle protein synthesis^61,62^ could have been responsible in explaining why no significant changes in muscle mass were observed.

Despite the reduction in fat content, this study did not observe significant increases in total body muscle mass in the resistance training group, contrary to pre-experimental assumptions and findings in existing literature. This may be attributed to the following factors:

1. **Dietary plan**: The diet plan developed by nutrition experts focused primarily on fat loss, emphasizing energy intake control. To maintain uniformity across the three exercise groups, protein intake was not specifically increased for the resistance training group. Studies have shown that, for effective muscle quality management in older adults, weight reduction of 0.5–1.0 kg per week or 8–10% of initial body weight over six months, combined with a daily protein intake of 1 g per kilogram of body weight and adequate micronutrient intake, is more effective^63,64^.
2. **Participant characteristics**: Muscle growth is influenced by age, gender, and training experience. Welle et al. found that the muscle hypertrophy response was lower in older adults compared to younger individuals after three months of resistance training^65^. The participants in this study were predominantly over 35 years old, surpassing the peak age for muscle mass development (18–25 years for men, 16–20 years for women). Moreover, more than 90% of participants had no prior experience with regular resistance training, requiring additional time to activate muscles and master proper techniques, which limited muscle growth within the 12-week.
3. **Training intensity and implementation**: The resistance training protocol was designed with a focus on safety and gradual progression, considering the participants’ exercise history, risk of injury, and the feasibility of home-based exercises. Self-weight and elastic band-assisted training were used at moderate intensity to activate target muscle groups and burn calories. However, this intensity was insufficient for significant muscle hypertrophy.

### Implementation and development of the “offline + online” mode of resistance training

The results of this study reveal that the hybrid online + offline training model significantly enhances adherence, engagement, and physiological outcomes compared to a dietary-only. Group R, which implemented resistance training, achieved the highest weight reduction and body fat percentage decrease, aligning with findings from studies such as Wewege et al. (2021), which highlighted the efficacy of resistance training in improving body composition when coupled with a supervised program^66^. Similarly, Groups F and H, focusing on aerobic and interval training, respectively, demonstrated substantial improvements, consistent with findings by Ouerghi et al. (2019), where combined training modalities enhanced adherence and cardiovascular health in hybrid models^67^. The high adherence rates and engagement scores in the present study underscore the value of real-time feedback and tailored programs, corroborating studies by Viana et al. (2020), which emphasized the importance of interaction with trainers to sustain participant motivation and achieve outcomes^68^.

Group C, on the other hand, exhibited minimal and statistically insignificant changes in body weight and fat percentage, similar to findings in purely dietary program studies^69^, which reported limited body composition improvements without exercise integration. Dietary-only programs have shown limited effectiveness in significantly altering body composition and weight, particularly when compared to integrated approaches. Studies like those by Brown et al. (2009) highlight moderate weight changes, emphasizing improved outcomes when combined with behavioral strategies^70^. Research by Lopes et al. (2021) underscores the challenges of achieving significant results without adherence and combination strategies^71^, while Chao et al. (2021) highlight the short-term benefits of low-calorie diets but the difficulty in maintaining long-term changes^72^. The results suggest that combining offline sessions to build foundational skills with online platforms for continuity can optimize outcomes, particularly when training is personalized. This study reaffirms the growing body of evidence advocating for hybrid training approaches to promote long-term health improvements, addressing barriers such as accessibility and participant engagement. Future research could further explore the scalability and effectiveness of such models across different populations.

### Limitations of the study

The study has several limitations that may influence the interpretation of its findings. The study population consisted exclusively of middle-aged individuals with obesity meeting China’s obesity criteria, restricting the applicability of the findings to other age groups or populations. Dietary adherence relied on self-reported data through an app, introducing potential reporting bias. Moreover, potential differences in protein intake between groups were not accounted for in this study. Uneven group sizes (ranging from 16 to 22 participants) may have impacted the statistical power of comparisons. Conducting over 50% of the sessions online introduced variability in supervision and performance compared to fully offline training. Additionally, participants self-selected their preferred exercise type rather than being randomly assigned. This self-selection brings about possibility of selection bias. Gender-specific differences were not fully explored, and the study primarily focused on physiological outcomes, overlooking psychological or behavioral factors such as motivation and perceived barriers. Future research should address these limitations to enhance the robustness of the findings.

### Conclusions and recommendations

Long-term resistance training significantly improves weight management, posture, and body composition in middle-aged individuals with obesity. Compared to maximal fat oxidation training and high-intensity aerobic interval training, resistance training offers greater benefits in reducing WHR in men. The “offline + online” training model proves to be both practical and safe, particularly during a time when COVID-19 remains a public health concern. This approach minimizes infection risks while effectively promoting fat loss through exercise.

Future research should focus on optimizing and innovating resistance training programs for middle-aged individuals with obesity. Emphasis should be placed on increasing dietary protein intake, precisely tailoring training loads, and enhancing muscle content through carefully designed resistance training protocols. Resistance training plays a critical role in maintaining muscle mass, improving muscle strength, and reducing the risk of osteoarthropathy and cardiovascular disease in middle-aged individuals. To maximize benefits, a two-phase training approach is recommended:

1. **Phase 1**: Focus on fat loss through resistance and aerobic exercises combined with caloric control.
2. **Phase 2**: Implement muscle-building training with progressively higher intensity, supported by a diet plan that includes increased protein intake.

This approach can enhance exercise capacity, promote muscle hypertrophy, and provide sustainable health benefits.

## Data Availability

All data is included in this manuscript.
